# S100A8/A9 in herpes zoster neuralgia: molecular mechanisms and therapeutic perspectives

**DOI:** 10.3389/fimmu.2025.1615638

**Published:** 2025-07-04

**Authors:** Dongzhi Jiang, Ziyuan Zhang, Fei Ren, Wenqin Sun

**Affiliations:** ^1^ Department of Anesthesiology, Changde Hospital, Xiangya School of Medicine, Central South University (The First People’s Hospital of Changde City), Changde, Hunan, China; ^2^ Xiangya Hospital, Central South University, Changsha, Hunan, China; ^3^ Department of Anesthesiology, Xiangya Hospital of Central South University, Changsha, Hunan, China

**Keywords:** S100A8/A9, neuroinflammation, pain transformation, herpes zoster, postherpetic neuralgia

## Abstract

Herpes zoster (HZ), a neurological disorder resulting from varicella-zoster virus (VZV) reactivation, presents significant clinical challenges, particularly in the development of postherpetic neuralgia (PHN). This comprehensive review elucidates the pivotal role of S100A8/A9, a damage-associated molecular pattern (DAMP) molecule, in the pathogenesis of chronic pain transition. By systematically analyzing molecular mechanisms, we provide unprecedented insights into the complex neuroimmune interactions underlying HZ-associated neuropathic pain. Our investigation reveals S100A8/A9’s critical function in modulating neuroinflammatory processes through TLR4/TNF signaling pathways, demonstrating its potential as a sophisticated therapeutic target. The protein complex orchestrates intricate cellular communications, fundamentally disrupting neuronal homeostasis and contributing to pain chronification. Emerging technological platforms, including single-cell sequencing and multi-omics analysis, have substantially enhanced our understanding of these molecular dynamics. The review critically examines current therapeutic limitations and proposes innovative research directions. By integrating molecular, cellular, and clinical perspectives, we highlight the urgent need for personalized intervention strategies that address the multifaceted mechanisms of HZ-induced neuropathic pain. Our findings underscore S100A8/A9’s significance as a promising biomarker and potential therapeutic intervention in neurological disorders.

## Introduction

Herpes zoster (HZ), resulting from varicella-zoster virus (VZV) reactivation, represents a complex neurological disorder characterized by postherpetic neuralgia (PHN) as its most debilitating sequela (1). Epidemiological data from 2021 reveal a global incidence of 711.5 per 100,000 population, with a marked age-dependent increase in both incidence and disease burden, particularly among individuals over 70 years old (2).

Current therapeutic interventions for HZ-associated pain exhibit limited efficacy, with approximately 40% of patients demonstrating poor treatment response, underscoring the urgent need to elucidate pain chronification mechanisms (3). Damage-associated molecular patterns (DAMPs), particularly the S100A8/A9 calcium-binding proteins, have emerged as critical neuroinflammatory mediators in chronic pain development (4). These proteins significantly modulate inflammatory responses through TLR4 and RAGE signaling pathway activation, playing a pivotal role in neuropathic pain progression (5). Despite extensive investigations of S100A8/A9 as an inflammatory biomarker, its specific potential in predicting postherpetic neuralgia remains largely uncharacterized (6).

Significant knowledge gaps persist regarding S100A8/A9’s role in pain chronification, including incomplete comprehension of its expression regulation, inter-patient variability, and limited clinical evidence supporting targeted therapeutic strategies (7). Emerging technological platforms, such as single-cell sequencing and multi-omics analysis, have substantially enhanced our understanding of S100A8/A9’s mechanistic involvement in neuro-glial interactions and neurological pathogenesis, providing critical theoretical foundations for potential innovative therapeutic approaches (8).

## Expression characteristics of S100A8/A9 during Varicella-Zoster Virus infection

S100A8/A9, as pivotal members of the calcium-binding protein family, orchestrate critical roles in diverse physiological and pathological processes, with particular prominence in pathogen-induced immune responses (9). Their dynamic expression patterns serve as sophisticated biomarkers across varied infectious scenarios, exhibiting remarkable significance in viral infections such as herpes zoster.

In herpes simplex virus-1 (HSV-1) experimental models (10), S100A9 demonstrates a distinctive upregulation within dorsal root ganglia, characterized by peak expression occurring 3–7 days post-infection and potentially persisting for up to 21 days. This temporal expression profile demonstrates remarkable correlation with postherpetic neuralgia progression.

Neuroinflammatory contexts reveal elevated calprotectin (S100-A8 and S100-A9) levels in cerebrospinal fluid among patients with HSV-1-induced meningitis or encephalitis (11). Proteomic investigations have uncovered nuanced protein dynamics: significantly elevated S100A9 levels were observed in herpes zoster (HZ) patients compared to healthy controls during early disease stages (duration < 3 months). Conversely, in post-herpetic neuralgia (PHN) patients (disease duration > 3 months), S100A8 exhibited significant upregulation relative to healthy volunteers. Notably, no statistically significant differences emerged in S100A9 or S100A8 levels between PHN and HZ patient cohorts (12).

Viral infections initiate intricate immunological cascades wherein S100A8/A9 function as critical inflammatory mediators. During influenza A virus infection, these proteins activate toll-like receptors through molecular pattern recognition, facilitating pro-inflammatory signaling via DDX21-TRIF pathways (13). Analogous inflammatory responses are documented across diverse pathogenic challenges, with HIV-1 infection demonstrating correlative elevations in S100A8/A9 levels, reflecting disease progression and immunological deterioration (14). Comparable protein expression dynamics are detected in bacterial infections, encompassing sepsis, typhoid fever, and pneumonia (15–17).

Remarkably, S100A8/A9 expression transcends traditional immune cell boundaries. Keratinocytes and non-classical immune cells substantially contribute to protein release during inflammatory processes, illustrating the intricate cellular mechanisms underlying host immune responses.

The immunoregulatory significance of S100A8/A9 proteins emerges from their capacity to modulate immune responses with exquisite precision. Their expression levels represent a sophisticated equilibrium between effective pathogen defense and potential inflammatory damage (18, 19). Both excessive and insufficient protein concentrations can disrupt immune homeostasis—either precipitating destructive inflammatory cascades or compromising host defense mechanisms. This delicate balance underscores the nuanced immunomodulatory strategy inherent in S100A8/A9 protein regulation.

## Cellular origin of S100A8/A9

S100A8/A9 represents a prominent cytosolic protein complex characterized by predominant expression within myeloid cell lineages. This heterodimeric protein is primarily localized in diverse immune cells, including neutrophils, monocytes, dendritic cells, myeloid-derived suppressor cells, and activated macrophage populations, with platelets and megakaryocytes further contributing to its cellular source (20).

Quantitative analyses reveal that S100A8/A9 constitutes approximately 40% of the total cytoplasmic protein content in circulating human neutrophils. Interestingly, beyond traditional myeloid compartments, non-myeloid cells such as vascular smooth muscle and endothelial cells demonstrate heightened S100A8/A9 accumulation under inflammatory or infectious stimuli (20).

The protein’s distribution extends beyond cellular environments, with free/soluble S100A8/A9 exhibiting a reference range of 1–6 mg/L in healthy individuals. During active inflammation, serum levels can increase up to 100-fold, although gender does not significantly influence these concentrations (21, 22). Its compact molecular structure facilitates diffusion between tissues and blood, enabling detection in various biological fluids including urine, body secretions, intestinal fluid, and feces, with fecal calprotectin typically ranging from 10–50 mg/L (21, 22).

Neutrophils emerge as the primary cellular source of S100A8/A9, as substantiated by comprehensive immunohistochemical investigations. Viral infection models, particularly HSV-1, demonstrate significant neutrophil infiltration in dorsal root ganglia (DRG) with pronounced S100A9 expression (18, 21). As calcium-binding proteins within the S100 family, S100A8 and S100A9 predominantly exist as heterodimers, reflecting enhanced structural stability.

These proteins function as sophisticated cellular calcium sensors, participating in critical physiological processes including cytoskeleton rearrangement and arachidonic acid metabolism. During inflammatory responses, S100A8/A9 is actively released, orchestrating complex immunomodulatory mechanisms by stimulating leukocyte recruitment and inducing cytokine secretion (9, 23). Under physiological conditions, sufficient S100A8/A9 stores exist in neutrophils and myeloid-derived dendritic cells, with baseline levels typically low in monocytes.

Infection-induced inflammatory processes represent a significant trigger for S100A8/A9 expression. In HSV-1 infected mouse models, S100A9 demonstrates a distinctive expression profile characterized by significant mRNA and protein level elevations in dorsal root ganglia. The expression peaks at 3–7 days post-infection and can be sustained for up to 21 days, notably correlating with postherpetic neuralgia development (18).

The pathogenesis of inflammatory conditions involves complex cellular dynamics. Persistent neutrophil activation and an elevated macrophage M1/M2 ratio substantially contribute to the progression of postherpetic neuralgia following herpes zoster infection (24). Moreover, tissue-specific expression patterns, such as those observed in intestinal epithelial cells, demonstrate intricate associations with inflammatory damage (25).

## Molecular mechanisms mediated by S100A8/A9 in pain chronification

### Receptor-mediated signaling pathways

S100A8/A9 employs complex receptor-mediated mechanisms to significantly contribute to pain chronification, primarily through interactions with Toll-like receptor 4 (TLR4) and receptor for advanced glycation end products (RAGE). Extensive research has demonstrated that these receptors mediate critical signal transduction and amplification processes in herpes zoster-associated neurological pathogenesis (26).

Following Herpes Simplex Virus-1 (HSV-1) infection, TLR4 expression is markedly upregulated in dorsal root ganglia (DRG). The binding of S100A9 to TLR4 triggers tumor necrosis factor (TNF) production, a pivotal mediator in herpes zoster neuralgia development (18, 27). Macrophage activation in DRG subsequent to Varicella-Zoster Virus (VZV) infection leads to the release of inflammatory factors such as High Mobility Group Box 1 (HMGB1), further promoting pain generation through RAGE and CXCL12/CXCR4 signaling pathways (28).

**Figure 1:**
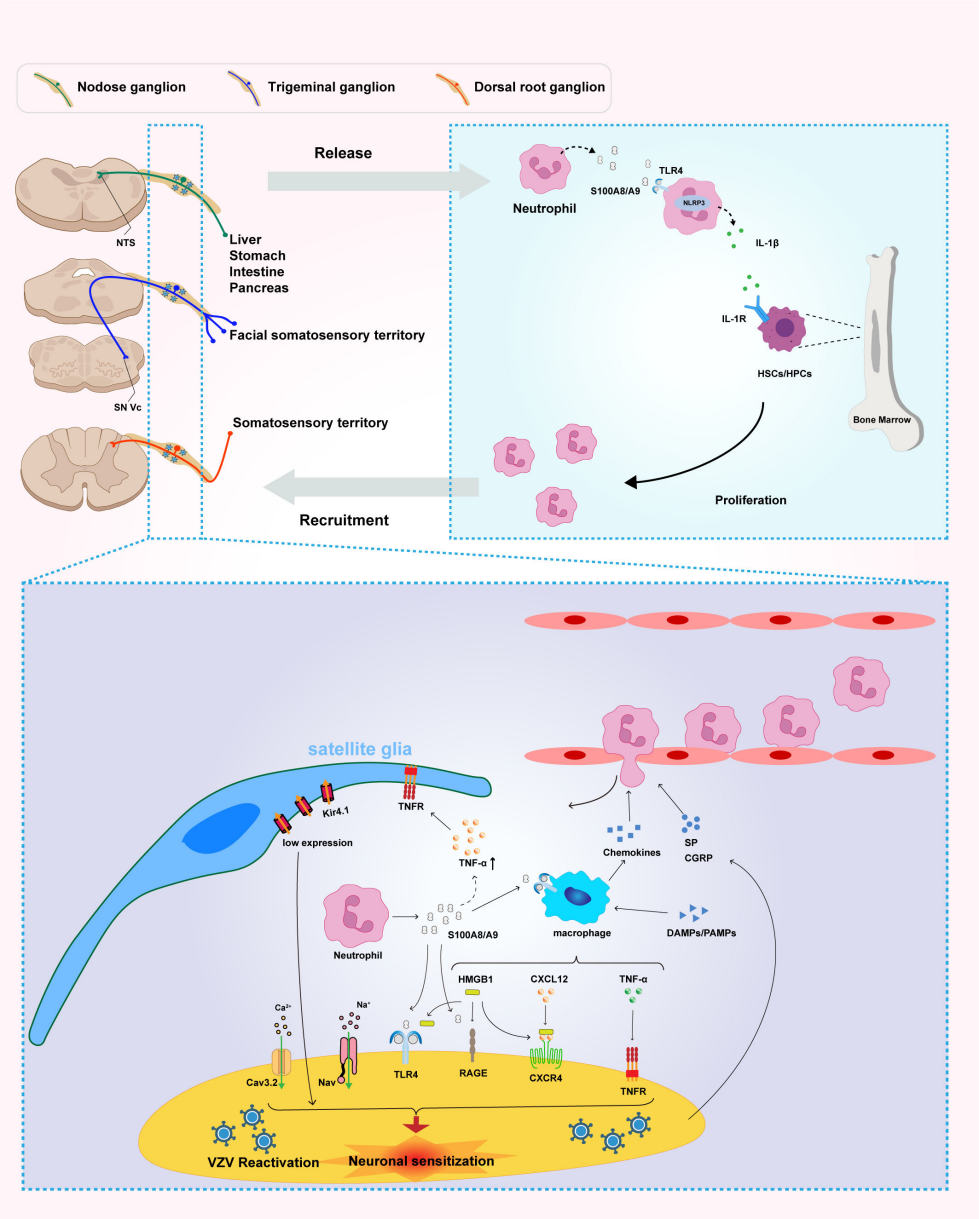
Neutrophil-derived S100A8/A9 mediates peripheral immune activation and neuroinflammation, contributing to pain sensitization and inflammatory amplification in postherpetic neuralgia (PHN). Reactivation of varicella-zoster virus (VZV) in sensory ganglia—including the nodose ganglion, trigeminal ganglion, and dorsal root ganglion (DRG)—triggers neutrophil infiltration and the subsequent release of S100A8/A9. This damage-associated molecular pattern (DAMP) activates the TLR4/NLRP3 inflammasome signaling pathway, thereby promoting the production and secretion of interleukin-1β (IL-1β). In turn, IL-1β acts on IL-1 receptor (IL-1R)-expressing hematopoietic stem and progenitor cells (HSCs/HPCs) within the bone marrow, enhancing granulopoiesis and establishing a systemic positive feedback loop of neutrophil generation and recruitment that sustains and amplifies systemic inflammation. At the local level, S100A8/A9 released from infiltrating neutrophils acts on multiple cell types within the microenvironment of sensory ganglia—including the nodose ganglion, trigeminal ganglion, and DRG—initiating neuroinflammatory processes. This molecule directly targets satellite glial cells (SGCs), macrophages, and sensory neurons, promoting inflammation and neuronal sensitization. Within neurons, both S100A8/A9 and HMGB1 function as DAMPs that bind to both TLR4 and RAGE receptors, triggering downstream excitatory signaling cascades. In addition, S100A8/A9 stimulates macrophages to produce tumor necrosis factor-alpha (TNF-α) and enhances the CXCL12–CXCR4 signaling axis, further amplifying local inflammatory responses. Importantly, S100A8/A9-induced upregulation of TNF-α activates SGCs through TNF receptors (TNFR), leading to downregulation of the Kir4.1 potassium channel, disruption of ionic homeostasis, and subsequent neuronal hyperexcitability and persistent pain. Multiple signaling pathways within sensory neurons—including Cav3.2, Nav, TLR4, RAGE, TNFR, and CXCR4—act in concert to mediate the transmission and maintenance of nociceptive signaling. Collectively, these findings highlight the central role of S100A8/A9 in the pathogenesis of PHN and identify its associated signaling network as a promising therapeutic target. Multiple signaling pathways within DRG neurons—including Cav3.2, Nav, TLR4, RAGE, TNFR, and CXCR4—cooperatively mediate the transmission and maintenance of nociceptive signals. Collectively, these findings underscore the central role of S100A8/A9 in the pathogenesis of PHN and highlight its signaling network as a promising target for therapeutic intervention.

The molecular cascade initiated by S100A8/A9 involves activation of MAPK and NF-κB signaling pathways, resulting in pro-inflammatory factor production and subsequent neuroinflammation exacerbation (18). The RAGE signaling pathway plays a particularly critical role in pain chronification, with S100A8/A9 binding triggering calcium-dependent signal transduction that profoundly affects intracellular calcium homeostasis and associated signaling networks. This mechanism demonstrates intimate associations with pain sensitization, especially in neuron-glia interactions (**Figure 1**).

## Calcium ion channel dynamics

Varicella-Zoster Virus fundamentally disrupts neuronal calcium channel dynamics, systematically modifying voltage-dependent and ligand-activated channel expressions. The virus alters calcium ion distribution within infected cells, significantly increasing intracellular calcium content. Specific modulation of the Cav3.2 T-type calcium channel demonstrates the virus’s capacity to disrupt neuronal calcium signaling homeostasis (29, 30).

This viral-induced calcium dysregulation represents a fundamental driver of persistent neuropathic pain mechanisms. Contemporary pharmacological interventions, such as gabapentin and pregabalin, target α2–δ voltage-gated calcium channel subunits but provide only partial symptomatic relief. Their limited efficacy stems from addressing singular calcium channel subtypes, failing to comprehensively interrupt the complex calcium signaling networks triggered by viral neuroinvasion (17) (**Figure 1**).

## Neuro-immune-glial cell interactions

Neuroinflammation emerges as a critical determinant in herpes zoster pain chronification, with S100A8/A9 serving as a pivotal inflammatory mediator regulating intricate interactions between nervous, immune, and glial systems (15, 18). Dorsal root ganglion (DRG) neurons represent a crucial junction in the neuro-immune interface, containing abundant stores of immunomodulatory neuropeptides, particularly calcitonin gene-related peptide (CGRP) and substance P (31–33). These neuropeptides are co-localized within specialized subsets of peptidergic nociceptive primary afferent neurons and serve as key mediators in neurogenic inflammation, orchestrating complex cellular communications through multiple mechanisms that contribute to pain chronification (34, 35).

## Neuropeptide release mechanisms and cellular dynamics

CGRP is abundantly expressed in trigeminal ganglion and DRG neurons, with release occurring from peripheral nerve terminals, central nerve terminals, and within the ganglion itself. Upon nociceptive stimulation or tissue damage, CGRP release is triggered through multiple convergent mechanisms, including direct depolarization-induced calcium-dependent exocytosis, inflammatory mediator-induced release by TNF-α and IL-1β, and S100A8/A9-mediated activation of MAPK and NFκB signaling pathways. Substance P, an 11-amino acid tachykinin, is co-released with CGRP from the same peptidergic neurons, creating a coordinated neuropeptide response that amplifies neurogenic inflammation (34, 35). This synchronized release of both neuropeptides establishes a synergistic inflammatory cascade that significantly enhances the magnitude and duration of the neurogenic inflammatory response, contributing to the development and maintenance of peripheral sensitization and chronic pain states.

The immunomodulatory functions of these neuropeptides are multifaceted and complementary. CGRP exerts potent vasodilatory effects through activation of the CGRP receptor complex (CLR/RAMP1/RCP), leading to increased cAMP levels, enhanced microvascular permeability, plasma protein extravasation, and nitric oxide synthesis upregulation (31). The neuropeptide demonstrates complex immunomodulatory properties, promoting mast cell degranulation with subsequent release of histamine and leukotrienes, influencing neutrophil adhesion and transmigration across endothelial barriers, and potentially promoting pro-inflammatory M1 macrophage polarization during acute inflammatory conditions. Concurrently, substance P binds to neurokinin-1 (NK1) receptors expressed on various immune and endothelial cells, inducing endothelial activation with enhanced expression of adhesion molecules (ICAM-1, VCAM-1, E-selectin, P-selectin), facilitating leukocyte migration and endothelial adhesion, and stimulating pro-inflammatory cytokine release (TNF-α, IL-1β, IL-6) (34). This initiates a self-perpetuating inflammatory cycle characterized by enhanced vascular permeability, recruitment of inflammatory cells to injury sites, and release of additional inflammatory mediators that further stimulate neuropeptide release.

## Bidirectional neuro-immune communication networks

The relationship between neuropeptides and immune mediators involves complex bidirectional signaling networks that perpetuate inflammatory responses. TNF-α directly stimulates CGRP expression and secretion from DRG neurons, creating positive feedback loops that maintain inflammation. IL-1β and IL-6 enhance neuropeptide synthesis and release while increasing receptor sensitivity, and IFN-γ modulates neuropeptide receptor expression on immune cells, altering their responsiveness to CGRP and substance P. Within the DRG microenvironment, CGRP and substance P released from neuronal cell bodies interact with surrounding satellite glial cells (SGCs), enhancing gap junction communication, promoting SGC activation with subsequent cytokine release, altering calcium signaling that amplifies neuronal excitability, and creating a local inflammatory microenvironment that promotes peripheral sensitization.

Neuropeptides significantly modulate immune cell dynamics through complementary mechanisms, with CGRP and substance P enhancing endothelial permeability and facilitating immune cell extravasation at sites of inflammation. These neuropeptides exert differential effects on T cell subsets, promoting Th1 and Th17 pro-inflammatory responses while potentially suppressing regulatory T cell (Treg) function, thereby shifting the immune balance toward enhanced inflammatory activity. Additionally, CGRP and substance P modulate T cell trafficking and tissue infiltration patterns, influencing the spatial and temporal distribution of immune responses (35). Both neuropeptides also interact with dendritic cells, affecting their maturation processes and antigen presentation capacity, which critically influences the transition from innate to adaptive immune responses. This multifaceted neuropeptide-mediated immune modulation establishes a neurogenic inflammatory environment that perpetuates and amplifies tissue inflammation, contributing to the chronification of inflammatory pain conditions.

## Glial cell activation and central sensitization mechanisms

Glial cell activation represents a primary pathway of S100A8/A9-mediated neuroinflammatory responses, significantly modulated by neuropeptide signaling. Satellite glial cell activation involves TNF generation, profoundly enhancing neuronal excitability (19), with CGRP and substance P amplifying this response through functional receptors expressed on SGCs, leading to enhanced inflammatory mediator production and altered calcium homeostasis. Microglial inflammatory polarization results in the release of multiple inflammatory factors, including TNF, CCL1, CCR7, CSF2, and CXCL9 (20), with CGRP and substance P directly influencing microglial activation states and potentially shifting the balance toward pro-inflammatory M1 phenotypes during acute inflammatory phases. Neuropeptide release further disrupts normal calcium homeostasis, with CGRP increasing intracellular cAMP levels and substance P altering calcium flux through NK1 receptor-mediated mechanisms, representing a crucial mechanism underlying pain generation and chronification (21).

Neuronal sensitization occurs through direct S100A8/A9 interaction with DRG sensory neuron peripheral axons, activating MAPK and NFκB signaling pathways that induce pro-inflammatory factor expression and release (22). This process is significantly amplified by autocrine and paracrine neuropeptide signaling, where released CGRP and substance P create positive feedback loops that maintain and enhance the sensitized state. The release of neuropeptides from central terminals in the dorsal horn represents a critical mechanism underlying central sensitization and chronic pain development. Upon noxious stimulation, CGRP and substance P released from primary afferent terminals facilitate synaptic transmission to second-order neurons through multiple convergent pathways (32, 33). These neuropeptides enhance glutamate release from presynaptic terminals and promote NMDA receptor phosphorylation, leading to increased calcium influx and enhanced synaptic efficacy. Concurrently, neuropeptide signaling triggers increased expression of immediate early genes, including c-fos and c-jun, which serve as molecular markers of neuronal activation and contribute to transcriptional changes associated with persistent pain states. The sustained neuropeptide release induces long-term potentiation-like changes in synaptic strength between primary afferents and dorsal horn neurons, resulting in enhanced responsiveness to subsequent stimuli and facilitating the recruitment of previously silent synapses, expanding the receptive fields of dorsal horn neurons and contributing to the spread of hyperalgesia beyond the initial site of injury.

## Therapeutic implications and future directions

The intricate neuropeptide-immune crosstalk presents multiple therapeutic intervention opportunities for inflammatory pain management. The clinical success of CGRP receptor antagonists and anti-CGRP monoclonal antibodies in migraine treatment demonstrates the therapeutic potential of targeting neuropeptide signaling pathways (32), suggesting similar approaches may be applicable to herpes zoster-related pain conditions and other neurogenic inflammatory disorders. Although NK1 receptor antagonists have shown limited success in chronic pain conditions, their established efficacy in chemotherapy-induced nausea and vomiting indicates potential applications in inflammatory pain states with significant neurogenic components (34). Furthermore, targeting multiple components of the neuropeptide-immune axis simultaneously through combination therapeutic approaches may provide enhanced efficacy, particularly in complex chronic pain conditions where multiple pathways are concurrently activated. This multifaceted targeting strategy could potentially address both the neuronal hypersensitization and immune-mediated inflammatory components that characterize chronic pain states, offering more comprehensive therapeutic solutions than single-target interventions.

The formation of the neuro-immune microenvironment represents a complex, bidirectional communication network significantly orchestrated by neuropeptide signaling. Sensory neurons promote CD8+ T cell activation and recruitment through neuropeptide and inflammatory factor release, while infiltrating immune cells influence neuronal functional states through cytokine release (18). The central role of CGRP and substance P in this process extends beyond simple neurotransmission to encompass comprehensive immunomodulation, involving direct effects on immune cell function, endothelial activation, and glial cell responses. This intricate molecular interplay underscores S100A8/A9’s critical role in bridging neurological and immunological processes, with neuropeptides serving as key amplifiers and modulators of the inflammatory response (15, 18). Understanding these mechanisms offers profound insights into chronic inflammatory pain mechanisms and provides a framework for developing more targeted therapeutic interventions that address both the neuronal and immune components of pain chronification (**Figure 1**).

## Clinical relevance and therapeutic perspectives

S100A8/A9 represents a promising biomarker and potential therapeutic target in inflammation-associated diseases. Preclinical investigations have demonstrated that pharmacological blockade of S100A8/A9 using small-molecule inhibitors or antibodies can improve pathological conditions in murine models. A comprehensive review emphasized its potential as a clinical biomarker and therapeutic intervention in sepsis and sepsis-induced organ injury (21).

Current research suggests targeted interventions focusing on the S100A8/A9-TLR4-TNF pathway may provide innovative strategies for herpes zoster neuralgia management. Dexamethasone and vitamin D show potential in alleviating neuralgia by stimulating S100A8 expression in macrophage cell lines, enhancing oxidative defense mechanisms during acute inflammation (9). Intrathecal dexamethasone treatment has specifically demonstrated efficacy in reducing HSV-1-induced mechanical hypersensitivity, with localized effects predominantly observed at the dorsal root ganglia (DRGs) level (27).

Inflammatory pathway modulation presents another promising therapeutic approach. HSV-1 peripheral infection triggers cyclooxygenase-2 (COX-2) upregulation in DRGs, and intrathecal administration of COX-2 inhibitor indomethacin effectively reduces herpetic pain hypersensitivity by suppressing COX-2 signaling pathways (27). The combined application of TNF inhibitors and antiviral drugs offers potential for both acute pain alleviation and postherpetic neuralgia (PHN) prevention.

Satellite glial cell (SGC) functional modulation emerges as a critical therapeutic strategy. TNF/TNFR1 signaling in SGCs plays a pivotal role in herpetic neuralgia development through Kir4.1 expression downregulation. Maintaining normal potassium channel function could provide novel approaches to PHN prevention and treatment (27).

Calcium channel pharmacotherapy represents an established intervention for neuropathic pain management. Medications like gabapentin and pregabalin inhibit hyperalgesia and central sensitization by binding to the α2-δ subunit of voltage-gated calcium channels (24). However, their partial efficacy underscores the need for more comprehensive therapeutic strategies.

The multifaceted role of S100A8/A9 in neuroinflammatory processes highlights its potential as a versatile therapeutic target. Future research directions should focus on developing targeted molecular interventions, exploring precise mechanisms of pain chronification, and designing comprehensive strategies that address multiple pathological mechanisms.

## Conclusions and perspectives

S100A8/A9 plays a pivotal role in the pathogenesis of herpes zoster neuralgia, demonstrating complex interactions within neuro-immune-glial systems. Through the TLR4/TNF signaling pathway, this protein complex participates in disease onset and progression, revealing intricate cellular communication mechanisms underlying neuropathic pain development.

Varicella-Zoster Virus infection induces profound alterations in calcium channel expression and signaling, representing a fundamental mechanism in herpes zoster neuralgia pathogenesis. These molecular perturbations fundamentally disrupt neuronal homeostasis, contributing to persistent pain chronification through sophisticated neuroimmunological interactions.

The multifaceted role of S100A8/A9 in neuroinflammatory processes underscores its potential as a promising therapeutic target. Current research has illuminated critical molecular pathways, providing unprecedented insights into the complex mechanisms of neuropathic pain generation and maintenance.

Future investigative efforts must prioritize developing precise, personalized therapeutic strategies. Multidisciplinary collaboration and technological innovations will be essential in translating fundamental scientific discoveries into effective clinical interventions. Comprehensive research should focus on elucidating detailed molecular interactions, developing targeted inhibition strategies, and exploring advanced treatment approaches.

While significant progress has been made in understanding S100A8/A9’s role in herpes zoster neuralgia, substantial challenges remain. Continued research is crucial to overcome current limitations and bridge the gap between scientific discoveries and transformative clinical treatments.
